# Barriers to breast cancer screening among female teachers: a qualitative study

**DOI:** 10.1186/s12889-025-23787-w

**Published:** 2025-08-08

**Authors:** Parvin Mangolian Shahrbabaki, Hossein Safizadeh, Narjes Amirzadeh, Mehrdad Shahi, Somayeh Zeidabadinejad

**Affiliations:** 1 Nursing Research Centre, Kerman University of Medical Sciences, Kerman, Iran; 2 Department of Community Medicine, School of Medicine, Research Center for Social Determinants of Health, Institute for Futures Studies in Health, Kerman University of Medical Sciences, Kerman, Iran; 3 Department of Pathology, Pathology and Stem Cell Research Centre, Afzalipour Faculty of Medicine, Kerman, Iran; 4 School of Medicine, Shiraz University of Medical Sciences, Shiraz, Iran; 5 Sirjan School of Medical Sciences, Sirjan, Iran; 6 Department of Anesthesiology, Sirjan School of Medical Sciences, Sirjan, Iran

**Keywords:** Breast cancer, Screening, Barriers, Qualitative study, Teachers

## Abstract

**Background:**

Early diagnosis of breast cancer is critical for effective treatment and improved prognosis. This study investigated the barriers to breast cancer screening among high school teachers in Kerman, Iran.

**Methods:**

In 2018, a qualitative design with purposeful sampling was used to recruit 35 high school teachers. Data were collected through semi-structured interviews and group discussions. Content analysis was conducted using MAXQDA 2020, and trustworthiness was ensured through triangulation and member checking.

**Results:**

Participants (mean age: 47 ± 6 years) reported low screening rates, with only 17.1% regularly performing breast self-examinations and 42.8% having undergone mammography. Identified barriers were grouped into individual/familial factors (e.g., low self-worth, procrastination, lack of knowledge, false confidence, emotional distress, fear, and unsupportive partners) and sociocultural factors (e.g., shame and limited access to facilities). These barriers underscore the need for targeted interventions to enhance screening participation.

**Conclusion:**

Multiple individual and sociocultural barriers hinder breast cancer screening among Iranian women. Improving awareness, implementing supportive policies, and empowering teachers may promote early detection and prevention efforts.

**Supplementary Information:**

The online version contains supplementary material available at 10.1186/s12889-025-23787-w.

## Plain language summary

The present qualitative study investigated the barriers to breast cancer screening among teachers in Kerman, Iran, to provide necessary context and accuracy. According to the research results, it seems that Iranian women faced many obstacles to breast cancer screening. Therefore, healthcare providers should inform the public about the role of different methods in the timely diagnosis and rapid treatment of breast cancer and encourage them to perform regular screening. The healthcare systems, with the help of the administrative system, should design interventional programs to change public attitudes and beliefs in regular breast cancer screening and support and train teachers, who play an important role in community health in the future.

**Supplementary Information:**

The online version contains supplementary material available at 10.1186/s12889-025-23787-w.

## Introduction

Breast cancer remains the leading cause of cancer-related deaths among women worldwide [1]. In 2022, it was the most frequently diagnosed cancer (11.6%), following lung cancer, with approximately 2.3 million new cases reported [2]. In Iran, studies indicate that the prevalence of breast cancer is over 23 cases per 100,000 individuals [3]. The average age of diagnosis is at least 10 years younger than in Western countries [4], with more than 50% of cases diagnosed before age 50 [5]. Timely diagnosis through effective screening can significantly improve survival rates and outcomes [6]. Screening refers to identifying disease prior to symptom onset or in those with early signs [7]. Effective cancer control programs not only improve survival but also reduce treatment costs and long-term care requirements [8]. Among cancer types, breast, cervical, colorectal, and oral mucosa cancers benefit most from early detection via screening. Available methods for breast cancer include breast self-examination, clinical examination, and mammography [9, 10]. Regular self-examination increases early detection and reduces mortality [11],while delays in diagnosis lead to disease progression, higher mortality, and lower survival rates [12]. Mammography, although the most reliable and commonly used technique, may still miss some tumors detectable by clinical examination [13, 14]. Nonetheless, it contributes to a 15–25% reduction in breast cancer mortality [15]. Despite its effectiveness, mammography may be overlooked, especially in developing countries, due to accessibility and perception issues [16]. In many such contexts, both healthcare providers and women do not fully prioritize screening programs [17, 18]. In Iran, 70% of women are diagnosed at advanced stages owing to the absence of structured educational or screening programs [19].Breast cancer screening in Iran is predominantly opportunistic, lacking a population-based mammography initiative. Mammograms are generally performed based on physician recommendation rather than a national program [20]. Although biennial mammography for women aged 50–70 is cost-effective, many Iranian women are diagnosed earlier [21]. Awareness campaigns rely heavily on mass media, and formal, systematized education efforts are limited [22]. Sociocultural taboos, limited access, and financial constraints result in low screening rates [20]. Reported barriers include time constraints, embarrassment, fear of diagnosis, absence of symptoms, and discomfort with male health professionals [23]. Structural challenges, such as navigating the healthcare system, lack of transportation, or distance to screening centers, also play a role [24, 25]. Meanwhile, strong doctor, patient relationships and clear health messages have the potential to improve public attitudes toward prevention and screening [26]. Although many researchers have attempted to elucidate the importance of screening, their efforts were constrained by methodological limitations. Predominantly, the studies tended to employ quantitative approaches that focused on isolated factors through predetermined hypotheses. In addition, We acknowledge that while several studies discuss breast cancer screening, most employ predominantly quantitative methods that do not fully explore women’s perceptions and the challenges they experience. For example, Gøtzsche and Nielsen (2011) as well as Marmot et al. (2013) critique the methodological limitations and narrow focus of many existing studies, thereby indirectly confirming that research specifically addressing women’s perceptions of screening behaviors and the difficulties encountered is limited [27, 28]. The sociocultural context of a society shapes individuals’ health-related beliefs and preventive behaviors. Among various social groups, teachers play a particularly influential role, their knowledge, attitudes, and behaviors significantly affect the health awareness of those around them, especially their students. However, studies show that teachers’ knowledge about breast cancer, particularly regarding risk factors and clinical signs, is generally inadequate [29, 30]. Given their prominent roles in both education and society, spanning urban centers to remote rural areas, female teachers in Iran serve not only as educators but also as influential and trusted advocates for community health promotion [30]. Despite this, few studies have examined the specific challenges they face regarding breast cancer screening. Therefore, this study aims to explore the unique context, barriers, and contributions of female teachers in Kerman, providing insights relevant to both the Iranian educational system and public health infrastructure.

## Materials and methods

### Sample 

This qualitative study used purposeful sampling to select Kermani teachers who met the inclusion criteria, such as teachers working in different high schools in Kerman who had three to five years of experience, had no mental illnesses, and could communicate and speak Persian. We tried to observe the maximum diversity among the participants in this study. To ensure maximum diversity, participants were selected from various school types (public and private) and different socioeconomic backgrounds. We also considered variations in age, gender, educational level, and personal experience with breast cancer screening The saturation of concepts and no acquisition of new data determine the end of sampling in qualitative studies [31]. The sampling in this study continued until data saturation was reached. Finally, 35 teachers were divided into groups of five to seven individuals. The study setting was high schools located in different districts of Kerman. We recorded the demographic characteristics of the participants at the beginning of each session, such as age, family history of breast cancer, history of clinical exam, regular breast self-examination, and mammography.

### Data collection

We conducted several group discussions, each lasting approximately 45–90 min, depending on the level of participant engagement. A semi-structured approach was employed, balancing adaptability with a clear focus on key themes. The researcher played a pivotal role in guiding the sessions, introducing topics, fostering meaningful dialogue, ensuring equitable participation, and preventing any undue influence. Additionally, careful attention was given to nonverbal cues to encourage interaction and maintain a dynamic yet constructive exchange of ideas. With participants’ consent, all discussions were meticulously recorded for further analysis, preserving the richness of the conversations.

### Data analysis

While individual interviews could provide personal insights, we opted for group discussions as they were more cost-effective, flexible, and conducive to stimulating recall and deeper reflection among participants. This approach encouraged dynamic interaction, allowing participants to build on each other’s thoughts, leading to enriched data and collective consensus rather than isolated individual opinions. Group discussions have proven effective in examining health behaviors and addressing health-related concerns, making them a valuable method for this study.

To facilitate discussions, we arranged seating in a semicircular form, invited participants, and provided reminders a day in advance. Clear ground rules were established at the beginning to encourage open and respectful dialogue. Initially, general questions were posed to create a comfortable environment, followed by more targeted inquiries focused on barriers to breast cancer screening. Participants were encouraged to engage freely, avoiding external influence or judgment, while nonverbal cues were carefully observed.

The structured questions included:

- How do you perceive breast cancer and its impact on women’s health?
- What preventive measures have you taken for early detection?
- What personal or societal barriers prevent women from undergoing regular screenings?
- How does cultural perception influence breast cancer screening behaviors?
- What role do healthcare providers and educational institutions play in promoting screening?
- Have you faced logistical challenges such as cost, accessibility, or availability of screening centers?
- How do psychological factors, such as fear or anxiety, affect your willingness to undergo screening?

Based on participants’ responses and previous analyses, supplementary questions were introduced to further explore underlying challenges. With consent, all discussions were recorded digitally and converted into files for detailed analysis.

### Trustworthiness and rigor

Data were analyzed using the qualitative content analysis approach developed by Lundman and Graneheim [32]. For this purpose, each interview was transcribed verbatim, was read several times for a general understanding of the content, primary codes and meaning units were determined, and the codes were merged and classified into more comprehensive categories based on similarity. Finally, the concept and content hidden in the data were extracted [33]. During data collection and analysis, the researcher wrote down her ideas related to the data and used them for subsequent interviews [34].To ensure data trustworthiness, the researcher engaged deeply with the research topic, data, and participants through prolonged interaction and in-depth interviews. Member checking was conducted by having participants review excerpts and initial codes for validation. Data management was carried out using Word and MAXQDA2020, ensuring systematic coding and organization. The entire dataset was thoroughly reviewed multiple times, analyzed verbatim, and coded to maintain accuracy. Additionally, peer debriefing sessions were held with supervisors and qualitative research experts to enhance the credibility and confirm the appropriateness of identified themes. Triangulation was employed by comparing findings across different sources and perspectives, strengthening the reliability of the results.

This study was reported in accordance with the COREQ (Consolidated Criteria for Reporting Qualitative Research) checklist (Tong et al., 2007).

## Results

We conducted this study on teachers working in Kerman high schools. The mean age of the participants was 47 ± 6 years; 33 were married (94.2%), 30 had bachelor’s and master’s degrees (85.7%), six participants (17.1%) experienced regular and accurate breast self-examination, 16 (45.7%) experienced clinical exams, and 15 (42.8%) got a mammogram. We identified three main categories of barriers to breast cancer screening (Table 1):

1. Individual and Familial Barriers: These include psychological and behavioral factors such as low self-worth, procrastination due to personal or work-related responsibilities, lack of knowledge about screening methods, misplaced confidence in personal health, emotional distress, fear stemming from previous experiences, and the influence of emotionally unstable partners.
2. Healthcare Provider Influence: Some barriers arise from the influence of medical professionals, for example, when the misinformation or specific opinions provided by healthcare providers discourage screening.
3. Sociocultural Barrier**s**: These include external factors, such as cultural shame that prevents open discussions about breast health, and structural limitations, such as insufficient screening facilities, restricted access, and long waiting times.

**Table 1:** Barriers to breast cancer screening and related subcategories

Barrier Category	Subcategories
Individual and Familial Barriers	Low self-worth-Procrastination and time constraints-Competing responsibilities-Limited awareness of screening methods-Perceived immunity and false confidence-Emotional distress and lack of motivation-Fear and negative past experiences-Lack of spousal support and emotional instability
Healthcare Provider Influence	Misinformation and physician guidance
Sociocultural Barriers	Cultural stigma and discomfort in screening-Limited access to screening facilities and environmental influences

### Individual and familial factors

#### Low self-worth

Prioritizing the health of family members, particularly that of children, was an obstacle that rendered a person’s own health unimportant.

“Kermani women are more concerned about their family members rather than themselves; they tend to forget about their own well-being.” (Participant #12)

“Our children, husbands, and even our parents are more important to us; we feel a heavy responsibility for our children that makes us place ourselves last.” (Participant #25)

#### Procrastination and time management issues

Participants expressed difficulties in prioritizing their health due to competing responsibilities and poor time management. Many admitted postponing breast cancer screening, not necessarily out of laziness, but because of a lack of structured health planning.

“I may visit a doctor in the future, but I keep postponing it because I struggle to find time.” (Participant #10)

“The biggest challenge for me is constantly delaying screening, I often forget due to my busy schedule.” (Participant #22)

#### Time constraints and competing responsibilities

Participants reported that their demanding schedules significantly limited their ability to prioritize breast cancer screening. As teachers, they often struggled to find time amid work responsibilities, household duties, and childcare obligations.

“I have a small child, and between family commitments and work, scheduling a screening feels impossible.” (Participant #14).

“I plan to go for screening, but with school in the morning and home responsibilities in the evening, I can never fit it in.” (Participant #28).

#### Limited awareness of breast cancer screening methods

Participants demonstrated a lack of knowledge regarding different screening approaches, leading to misconceptions about early detection and preventive measures. Many women avoided breast self-examinations due to unfamiliarity with breast anatomy and difficulty distinguishing normal tissue from potential abnormalities. (No specific quote is retained here after reclassification).

#### Perceived immunity and false confidence

Many participants believed that maintaining a healthy lifestyle, exercising regularly, or having no family history of breast cancer eliminated the need for screening. This false confidence led them to avoid preventive measures, assuming they were not at risk.

“I have had 5–6 self-examinations in my life. I do not need screening when there is no problem.” (Participant #8)

“I have not gone for a clinical breast exam because I am sure that I am healthy. I think everyone is their own doctor and knows what is going on in their body.” (Participant #15)

“I never thought that this could happen to me one day, and I have never faced such an incident around me. I always think that cancer happens to others, not to me, so I do not screen at all.” (Participant #21)

#### Emotional distress and lack of motivation

Many participants identified mental health issues such as depression, anxiety, and emotional distress as significant barriers to breast cancer screening. Feelings of unhappiness and low life satisfaction contributed to the neglect of preventive health measures, as women struggling with emotional well-being often deprioritized their own needs.

“I think mental problems are one obstacle that prevents women from screening. Unfortunately, Iranian women do not have much vitality, happiness, or life expectancy.” (Participant #9) “Factors such as unhappiness and depression make us not take care of ourselves.” (Participant #20).

#### Fear and negative past experiences as screening barriers

Participants expressed deep fears regarding a breast cancer diagnosis and its potential consequences, including loss of hair and beauty due to chemotherapy, physical disability, and even mortality. Some women developed a cancer phobia after witnessing the experiences of others, making them hesitant to seek early detection. Additionally, painful past experiences with mammography reinforced avoidance behaviors.

“There is a misconception that a person with cancer will survive three or four years, which creates a cancer phobia.” (Participant #7)

“I underwent breast cancer surgery, but I was afraid, so there was a long time between the first time I touched the tumor and the time I went for screening.” (Participant #13)

“I got a mammogram eight years ago, which was so painful that I would never get it again.” (Participant #25)

#### Lack of spousal support and emotional instability

Participants frequently highlighted the role of family dynamics, particularly spousal attitudes, as a significant barrier to breast cancer screening. Concerns about losing physical attractiveness, emotional instability within relationships, and fear of abandonment were commonly cited. Many women felt discouraged from seeking medical help due to their husbands’ pessimism, insensitivity, or lack of emotional support, which led to heightened anxiety about their future. “I know men who did not support their wives and even left them when they found out about their wives’ cancer.” (Participant #19)

### Healthcare provider influence

#### Misinformation and physician guidance

Some screening decisions were shaped by the influence of healthcare providers. In particular, inaccurate or overly cautious guidance sometimes discouraged women from undergoing screening procedures.

“I am not going to get a mammogram, but I will get a pap smear because the doctor believes that mammography has a high level of X-rays,” stated Participant #17.

### Sociocultural factors

#### Cultural stigma and discomfort in screening

In Iranian society, cultural norms regarding modesty and gender interactions contribute to feelings of shame and discomfort when discussing or undergoing breast cancer screening, especially when male doctors are involved. Many women avoid clinical breast exams and mammography due to embarrassment, social taboos, and a reluctance to openly address their health concerns.

“Our daughters feel ashamed to ask about their body parts, and they should feel free to talk about their problems,” noted Participant #12.

#### Limited access to screening facilities and environmental influences

Participants noted that the lack of easily accessible screening centers and long waiting times for appointments are significant obstacles to breast cancer screening. Many expressed frustration over the difficulties in scheduling timely checkups, which discouraged them from seeking preventive care. Furthermore, environmental factors in Kerman, such as its arid climate and the absence of recreational spaces, contributed to feelings of low motivation and emotional distress, further impacting health-related behaviors.

“Kerman has a dry climate with rare rainfall, which makes us sad. You become cheerful when you see grass, flowers, plants, the sea, forests, and rain,” reported Participant #5.

“One reason could be the lack of screening centers. Predetermined appointments are very time-consuming,” stated Participant #16.

“The interactions are not appropriate, and the appointments are very bad. The lack of screening centers is an obstacle in itself. If there are many centers, we will easily screen for breast cancer,” explained Participant #22.

## Discussion

This study explored the barriers to breast cancer screening among female teachers in Kerman, emphasizing the interplay of individual, familial, sociocultural, and structural factors that shape screening behaviors. While previous research has identified similar barriers, including lack of awareness, emotional distress, fear, cultural stigma, and financial constraints, this study provides a more nuanced understanding of their impact within this specific population and highlights implications for intervention development.

### Individual and familial barriers

One of the key findings was the tendency of women to prioritize family responsibilities over personal health. This aligns with Lamieian et al. [35],who identified domestic responsibilities and reliance on spousal support as major deterrents to screening. Similarly, Mokhtari et al. [36] found that forgetfulness and procrastination, often driven by work and household obligations, significantly reduced screening rates among working women. Addressing this barrier requires interventions that integrate breast health education into workplace wellness programs and promote time-efficient screening methods tailored for busy professionals.

Lack of knowledge was another critical factor influencing screening behaviors, reinforcing findings from Secginli et al. [37], who reported that more than half of surveyed women over 40 had never encountered information on mammography. Without proper health education, misconceptions can flourish, leading to heightened fear and avoidance of screening. Public health initiatives must prioritize targeted awareness campaigns that equip women, especially educators, with accurate knowledge about breast cancer risks, screening guidelines, and preventive strategies.

False confidence also emerged as a barrier, with participants believing their healthy lifestyle choices rendered them immune to breast cancer. This misconception echoes Hafezpour’s study [38], in which poor health literacy led women to overestimate their well-being and neglect preventive measures. American-Korean women similarly exhibited this belief, identifying “not being at risk” as a dominant reason for avoiding mammography [39].Interventions must directly counteract these myths by emphasizing the multifactorial nature of breast cancer risk and integrating risk assessment tools into primary healthcare settings to encourage screening among asymptomatic individuals.

### Psychological and emotional barriers

Emotional distress, including depression and concerns about life expectancy, significantly influenced screening hesitancy, aligning with Hafezpour’s findings [38], which linked negative emotions to avoidance behaviors. Conversely, studies have shown that a positive psychological outlook fosters proactive health engagement, with early diagnosis associated with improved well-being [40, 41]. Public health programs must incorporate mental health support into screening initiatives, ensuring that psychological counseling is available to mitigate emotional resistance to screening.

Cancer phobia was another notable barrier, with participants expressing intense fear that a diagnosis would confirm their worst anxieties. Lamieian et al. [35] found that fear generated by healthcare providers deterred women from screening, while Mokhtari et al. [36] identified tumor-related fear as the leading reason for avoiding clinical breast exams. Interestingly, Shahvari et al. [40] observed that fear could sometimes act as a motivator, with witnessing loved ones battle cancer encouraging screening participation. Understanding this dual nature of fear is critical, interventions must address anxiety through reassuring messaging and peer-support programs that normalize screening rather than amplify fear.

### Sociocultural barriers

Cultural stigma surrounding breast health remained a significant sociocultural barrier. Calderón-Garcidueñas et al. [42] identified cultural shame as a primary reason for screening avoidance, while Vetto et al. [43] noted that ethnic biases discouraged women from seeking exams from male doctors. Mokhtari et al. [36] further reinforced that screening behaviors are shaped by sociocultural norms, highlighting the need for culturally sensitive outreach efforts.

Interestingly, participants in this study linked environmental factors, such as Kerman’s dry climate, to feelings of unhappiness and diminished motivation for preventive healthcare. This aligns with Azaiza and Cohen [44], who found that Jewish women engaged in screening more frequently than Arab women due to stronger cultural beliefs in health awareness. The Breast Health Global Initiative [45] has emphasized the importance of education and cultural adaptation in improving screening uptake, reinforcing the need for tailored interventions that address local sociocultural dynamics.

### Structural and financial barriers

Financial constraints and limited healthcare access were critical barriers affecting screening behaviors. Participants reported that high screening costs, insurance limitations, and inadequate screening facilities restricted their ability to seek preventive care. This is consistent with Lamieian et al. [35], who cited ineffective health campaigns, overcrowded hospitals, and limited screening availability as major contributors to low screening rates. Abuidris et al. [46] similarly highlighted challenges such as weak referral systems, a shortage of specialized personnel, and insufficient educational resources as impediments to screening.

Moreover, negative attitudes toward cancer prevention remain a widespread issue, even among highly educated women. Abuidris et al. [47] found that a lack of awareness and ingrained misconceptions led many women to disregard clinical breast exams and mammography. Intervention efforts must focus on improving accessibility by providing affordable screening services, expanding the availability of screening centers, and integrating structured breast health education into routine healthcare practices.

## Conclusions

This study revealed that Iranian women face multiple barriers to breast cancer screening, influenced by sociocultural, organizational, and personal factors. To improve participation rates, healthcare providers should actively inform the public about the importance of regular screening for early detection and treatment of breast cancer. Additionally, healthcare and administrative systems should collaboratively design and implement culturally sensitive intervention programs to shift attitudes and beliefs toward screening, while specifically empowering and educating teachers, key agents in promoting community health.

### Implications and recommendations

#### For teachers and schools

- Implement Targeted Training: Schools should organize professional development programs to enhance teachers’ knowledge and confidence in promoting health-related topics.
- Promote Health Literacy Events: Initiate annual school-based health days to raise awareness about breast cancer and early detection strategies.
- Encourage Dialogue: Conduct peer-led workshops to normalize open discussions on breast health and overcome social stigma.
- Provide Time-Sensitive Access: Establish designated time slots or leave policies for teachers to attend health screenings without disrupting their duties.

#### For researchers

- Investigate Cultural Norms: Further studies should explore the influence of traditional caregiving roles and societal expectations on screening behaviors among women.
- Assess Intervention Effectiveness: Evaluate the impact of community outreach programs, educational campaigns, and family support structures on screening participation.
- Conduct Occupational Comparisons: Compare teachers’ screening behaviors with those of other professions to identify job-specific barriers and motivators.

#### For the healthcare system

- Enhance Service Accessibility: Increase the number of screening centers and subsidize costs to encourage widespread utilization.
- Adopt Supportive Policy Measures: Develop national policies granting paid preventive medical leave for educators and other vulnerable groups.
- Launch Public Campaigns: Implement culturally tailored national health campaigns to dispel common myths and foster a proactive attitude toward regular breast cancer screening.

## Supplementary Information

Supplementary Material 1

## Data Availability

The datasets generated and/or analyzed during the current study are not publicly available due to privacy concerns and the sensitive nature of qualitative data. However, anonymized data may be obtained from the corresponding author upon reasonable request.
