# Supplementary material for: Barriers to breast cancer screening among female teachers: a qualitative study
Source: BMC Public Health. 2025 Aug 8;25:2703. doi: 10.1186/s12889-025-23787-w (PMC12333085; doi:10.1186/s12889-025-23787-w)
Supplement: Supplementary file 1 — Supplementary Material 1 [file 12889_2025_23787_MOESM1_ESM.docx]

**Consolidated criteria for reporting qualitative studies (COREQ): 32-item checklist**

**MANUSCRIPT TITLE:**

| **No. Item** | **Guide questions/description** | **Reported on Page #** |
| --- | --- | --- |
| **Domain 1: Research team and reflexivity** |  |  |
| *Personal Characteristics* |  |  |
| 1. Inter viewer/facilitator | Which author/s conducted the interview or focus group? | Interviews were conducted by Dr. Parvin Mangolian Shahrbabaki as part of the research process |
| 2. Credentials | What were the researcher’s credentials? (E.g.  PhD, MD) | Dr. Parvin Mangolian Shahrbabaki (Associate Professor, Nursing Research), Dr. Hossein Safizadeh (Community Medicine), Narjes Amirzadeh (Community Health Practitioner), Mehrdad Shahi (MD Student), and Somayeh Zeidabadinejad (M.Sc. in Critical Care Nursing) |
| 3. Occupation | What was their occupation at the time of the study? | Dr. Parvin Mangolian Shahrbabaki was an Associate Professor in Nursing Research. Dr. Hossein Safizadeh worked in Community Medicine and Social Determinants of Health. Narjes Amirzadeh was a Community Health Practitioner. Mehrdad Shahi was an MD student, and Somayeh Zeidabadinejad held an M.Sc. in Critical Care Nursing |
| 4. Gender | Was the researcher male or female? | Dr. Parvin Mangolian Shahrbabaki (female), Dr. Hossein Safizadeh (male), Narjes Amirzadeh (female), Mehrdad Shahi (male), and Somayeh Zeidabadinejad (female) |
| 5. Experience and training | What experience or training did the researcher have? | Dr. Parvin Mangolian Shahrbabaki, an Associate Professor in Nursing Research, has significant expertise in health interventions. Dr. Hossein Safizadeh specializes in community medicine and social determinants of health. Narjes Amirzadeh is a trained Community Health Practitioner. Mehrdad Shahi, an MD student, has clinical training in medical sciences. Somayeh Zeidabadinejad holds an M.Sc. in Critical Care Nursing, bringing expertise in patient care and intervention strategies |
| *Relationship with*  *participants* |  |  |
| 6. Relationship established | *Was a relationship established prior to study commencement?*  . | No prior relationship was established between the researchers and participants before the commencement of the study |
| 7. Participant knowledge of the interviewer | *What did the participants know about the researcher? (e.g. personal goals, reasons for doing the research).* | Participants were aware that the researchers aimed to explore barriers to breast cancer screening among female teachers and develop potential intervention strategies based on the findings |
| 8. Interviewer characteristics | *What characteristics were reported about the interviewer/facilitator? (e.g. Bias, assumptions, reasons and interests in the research topic)* | The interviewer maintained a neutral stance, ensuring that responses were gathered without bias. Their interest in the topic stemmed from a commitment to improving breast cancer screening accessibility and understanding behavioral barriers among female teachers |
| **Domain 2: Study design** |  |  |
| *Theoretical framework* |  |  |
| 9. Methodological orientation and Theory | *What methodological orientation was stated to underpin the study? (e.g. grounded theory, discourse analysis, ethnography, phenomenology, content analysis).* | This study utilized a qualitative approach, employing content analysis to systematically explore barriers to breast cancer screening among female teachers. Purposeful sampling was used to ensure maximum diversity in participant selection. Data were collected through semi-structured group discussions, allowing for dynamic interaction and deeper reflection. The analysis process continued until data saturation was achieved, ensuring a comprehensive understanding of the themes |

| *Participant selection* |  |  |
| --- | --- | --- |
| 10. Sampling | *How were participants selected? (e.g. purposive, convenience, consecutive, snowball)* | Participants were selected using purposive sampling, ensuring maximum diversity. Selection criteria included teachers working in various high schools in Kerman with three to five years of experience, no mental illnesses, and proficiency in Persian. The study aimed to include individuals from different socioeconomic backgrounds, school types (public and private), and varying levels of education and experience with breast cancer screening. Sampling continued until data saturation was achieved |
| 11. Method of approach | *How were participants approached? (e.g. faceto-face, telephone, mail, email)* | Participants were approached face-to-face in their respective high schools, ensuring direct interaction and clarity about the study objectives. This method facilitated engagement and allowed researchers to explain the study in detail, answer questions, and obtain informed consent effectively |
| 12. Sample size | *How many participants were in the study?* | The study included a total of 35 participants, divided into groups of five to seven individuals for data collection through group discussions |
| 13. Non-participation | *How many people refused to participate or dropped out? Reasons?* | No participants refused to participate or dropped out during the study. All selected individuals completed the group discussions as planned |
| *Setting* |  |  |
| 14. Setting of data collection | *Where was the data collected? (e.g. home, clinic, workplace)* | Data was collected in high schools across different districts of Kerman, ensuring a diverse representation of participants in a familiar and relevant setting |
| 15. Presence of nonparticipants | *Was anyone else present besides the participants and researchers?* | High schools were chosen as the study setting to ensure participants were in a comfortable and familiar environment, facilitating open discussions and accurate data collection |
| 16. Description of sample | *What are the important characteristics of the sample? (e.g. demographic data, date)* | Demographic data such as age, family history of breast cancer, previous clinical exams, breast self-examinations, and mammography history were recorded at the beginning of each session. The data collection process took place in high schools across different districts of Kerman over approximately three months, from March to the end of May 2019, ensuring a representative sample and diverse perspectives |
| *Data collection* |  |  |
| 17. Interview guide | *Were questions, prompts, guides provided by the authors? Was it pilot tested?* | A semi-structured interview guide was developed to maintain consistency while allowing flexibility in participant responses. The guide included key prompts focusing on barriers to breast cancer screening, personal experiences, and influencing factors. To enhance discussion quality, seating was arranged in a semicircular form, and participants received reminders a day in advance. Clear ground rules were established at the beginning to foster open and respectful dialogue. Initially, general questions were posed to create a comfortable environment, followed by more targeted inquiries. Nonverbal cues were carefully observed throughout discussions.  Prior to the main study, the interview guide was reviewed in a pilot test with a small participant group, ensuring clarity and relevance of questions. This process allowed researchers to refine the wording and structure of prompts to maximize engagement and data richness |
| 18. Repeat interviews | *Were repeat interviews carried out? If yes, how many?* | No repeat interviews were conducted. All data were collected during the initial group discussions |
| 19. Audio/visual recording | *Did the research use audio or visual recording to collect the data?* | Audio recording was used to document all group discussions with participants' consent. This ensured the accurate preservation of conversations for further analysis. No visual recording was conducted during the study |
| 20. Field notes | *Were field notes made during and/or after the interview or focus group?* | Field notes were taken during and after each group discussion to complement the recorded data. These notes captured nonverbal cues, key discussion points, and any relevant observations that could provide additional context for the analysis |
| 21. Duration | *What was the duration of the inter views or focus group?* | Each focus group discussion lasted approximately 45 to 90 minutes, depending on the level of participant engagement and the depth of discussion |
| 22. Data saturation | Was data saturation discussed? | Data saturation was considered a key criterion for determining the completion of sampling. The process continued until no new themes or concepts emerged from the discussions. Once saturation was reached, no additional participants were recruited, ensuring the depth and reliability of the collected data |
| 23. Transcripts returned | *Were transcripts returned to participants for comment and/or correction?* | Transcripts were not returned to participants for review. However, careful attention was given to accurately documenting their responses during the discussions |
| **Domain 3: analysis and findings** |  |  |
| *Data analysis* |  |  |
| 24. Number of data coders | How many data coders coded the data? | Data coding was conducted by a single researcher, ensuring consistency in identifying themes and subcategories |
| 25. Description of the coding tree | *Did authors provide a description of the coding tree?* | The authors did not explicitly provide a description of the coding tree. However, themes and subcategories were systematically identified and analyzed to ensure coherence in data interpretation |
| 26. Derivation of themes | *Were themes identified in advance or derived from the data?* | Themes were derived inductively from the collected data. Through qualitative analysis, recurring patterns and concepts emerged organically based on participant responses, ensuring that the findings reflected their lived experiences and perspectives |
| 27. Software | *What software, if applicable, was used to manage the data?* | No specific software was used to manage the data. The analysis was conducted manually through thematic coding and categorization |
| 28. Participant checking | *Did participants provide feedback on the findings?* | Participants did not formally review the findings. However, efforts were made to accurately represent their perspectives through direct quotations and thorough thematic analysis |
| *Reporting* |  |  |
| 29. Quotations presented | *Were participant quotations presented to illustrate the themes/findings? Was each quotation identified? (e.g. participant number)* | Participant quotations were provided to illustrate key themes and findings. Each quotation was explicitly attributed to a specific participant using identifiers (e.g., Participant #12, Participant #25), ensuring transparency and contextual clarity |
| 30. Data and findings consistent | *Was there consistency between the data presented and the findings?* | Findings were systematically developed based on participant responses, ensuring consistency between the raw data and thematic interpretations. The classification of barriers aligns with patterns observed in discussions, reinforcing the credibility of the analysis |
| 31. Clarity of major themes | *Were major themes clearly presented in the findings?* | Major themes were clearly presented in the findings, with distinct categories and subcategories identified. Each theme was systematically analyzed and supported by participant quotations, ensuring clarity and coherence in the reporting |
| 32. Clarity of minor themes | Is there a description of diverse cases or discussion of minor themes? | In addition to major themes, the study also explored minor themes that provided further insights into participants’ perspectives. Diverse cases and nuanced findings were discussed to ensure a comprehensive understanding of the barriers to breast cancer screening |
